# A national survey of admission practices for late preterm infants in England

**DOI:** 10.1186/1471-2431-14-150

**Published:** 2014-06-17

**Authors:** Paul F Fleming, Puneet Arora, Rebecca Mitting, Narendra Aladangady

**Affiliations:** 1 Neonatal Intensive Care Unit, Homerton University Hospital NHS Foundation Trust, London, UK; 2 Centre for Paediatrics, Barts and the London School of Medicine and Dentistry, Queen Mary University of London, London, UK; 3 Department of Paediatrics, SDM College of Medical Sciences & Hospital, Dharwad, India

**Keywords:** Late preterm infant, Low birth weight, Postnatal ward admission, Nursery nurse, Guideline

## Abstract

**Background:**

Infants born at 34^+0^ to 36^+6^ weeks gestation are defined as ‘late preterm’ infants. It is not clear whether these babies can be managed on the postnatal ward (PNW) or routinely need to be admitted to the neonatal unit after birth.

**Aim:**

To conduct a national survey of admission practice for late preterm and low birth weight infants directly to the PNW after birth in England.

**Methods:**

All neonatal units were identified from the Standardised Electronic Neonatal Database (SEND). Individual units were contacted and data collected on their admission practice.

**Results:**

All 180 neonatal units in England responded. 49, 84 and 47 Units were Special Care Units (SCUs), Local Neonatal Units (LNUs) and Neonatal Intensive Care Units (NICUs) respectively. 161 units (89%) had written guidelines in relation to direct PNW admission for late preterm infants.

The mean gestational age of infants admitted directly to the PNW was significantly lower in LNUs compared to SCUs and NICUs compared to LNUs. Mean birth weight limit for direct PNW admission was significantly lower in NICUs compared to SCUs.

72 units had PNW nursery nurses. There was no significant difference in gestational age or birth weight limit for direct PNW admission in the presence of PNW nursery nurses.

**Conclusions:**

Admission practices of late preterm infants directly to the PNW varies according to designation of neonatal unit in England. Further studies are needed to establish the factors influencing these differences.

## Background

Late preterm infants are defined as premature infants born between 34 + 0 and 36 + 6 weeks gestation [1]. Observational studies from the United States have previously shown that the incidence of late preterm births has grown substantially over the last two decades. In 2006 it was estimated that 8.1% [2] of all births were late preterm which represented about 70% of all preterm deliveries [1]. The exact cause for this rise has not been identified but increased maternal age and increased uptake of assisted reproduction therapies have been implicated.

While infants born before 32 weeks gestation represent those at greatest risk for short and long term morbidity and mortality, it is well recognised that infants born late preterm are also at increased risk of both acute and chronic complications. Acute problems include respiratory distress [3,4], metabolic disorders (including hypoglycaemia and jaundice) [5-7] and infection and feeding issues [8,9]. All of these factors may increase the length of initial hospital stay. Intermediate issues include increased rates of hospital readmission [10] and long term problems include an increased risk of adverse neurodevelopmental outcomes [11,12].

Some late preterm infants may be mature enough to be managed in settings similar to term infants but there is limited published outcome data for late preterm infants who are admitted directly to the postnatal ward for on-going care. Although it is accepted that some of these infants may go on and require admission to the neonatal unit, a proportion of well late preterm babies can be exclusively managed on the postnatal ward.

At present there are no national guidelines in England relating to postnatal ward care for late preterm infants. Anecdotally there is wide variation between centres in relation to which birth weight and gestation category infants are considered eligible for direct post natal ward admission.

The aim of this study was to establish whether individual units in England have direct postnatal ward admission guidelines in relation to late preterm infants and if so which birth weight and gestation category are used for guidance. We also sought to examine whether or not unit designation (level) and the presence of paediatric nurses (nursery nurses) on the post natal ward affected admission practices.

## Methods

This questionnaire based study was conducted between January and August 2010. All neonatal units in England were identified from the Standardised Electronic Neonatal Database (SEND). Individual units were contacted by members of the research team via telephone and either the senior nurse or a physician was questioned. If no one was available to speak to the research team a maximum of 2 follow-up calls were made.

The questionnaire comprised 5 questions. These included:

1. The unit designation (Special Care Baby Unit, Local Neonatal Unit or Neonatal Intensive Care Unit)

2. Whether or not there is a direct postnatal ward admission policy for late preterm infants

3. What is the gestation cut off for direct postnatal ward admission

4. What is the birth weight cut off for direct postnatal ward admission

5. Whether or not nursery nurses were present on the postnatal ward

Centres caring for new-born babies in England are designated into one of three categories based on nationally agreed guidelines [13] and include:

• Special care units (SCUs) which provide special care for their own local population. SCUs provide a stabilisation facility for babies who need to be transferred to a neonatal intensive care unit (NICU) for intensive or high dependency care.

• Local neonatal units (LNUs) which provide neonatal care for their own catchment population, except for the sickest babies. They provide all categories of neonatal care, but they transfer babies who require complex or longer-term intensive care to a NICU, as they are not staffed to provide longer-term intensive care. The majority of babies over 27 weeks of gestation will usually receive their full care, including short periods of intensive care, within their LNU.

• Neonatal intensive care units (NICUs) are sited alongside specialist obstetric and feto-maternal medicine services, and provide the whole range of medical neonatal care for their local population. Many NICUs in England are co-located with neonatal surgery services and other specialised services.

Data were entered to an EXCEL database and the results analysed descriptively. Continuous outcomes were compared using an unpaired student t-test. Comparison of means by hospital designation was done using a 1 way test of variance (ANOVA). All statistics were performed using GraphPad Prism 5 and GraphPad Quickcalcs (GraphPad Software, Inc. San Diego, CA, USA).

The Chair of the East London Research and Ethics Committee confirmed this study meets the National Research and Ethics Service guidance for service evaluation and as such formal ethics approval was not required.

## Results

There were 184 centres identified on SEND of which 4 were no longer commissioned for looking after babies at the time of this study. Of the 180 units remaining, all centres responded to the questionnaire giving a response rate of 100%.

Among the responders 49 were Special Care Units (SCUs), 84 were Local Neonatal Units (LNUs) and 47 were Neonatal Intensive Care Units (NICUs). 161 units (89%) had a written guideline in relation to direct postnatal ward admission for late preterm infants. Of the 18 units (10%) that did not, all responded that a verbal agreement exists locally. One responder did not know if a formal guideline existed.

Table 1 shows the mean (standard deviation) and median (range) birth weight and gestation used as a cut off for direct post natal ward admission given by responders. When units were compared by designation, significantly lower gestational age infants were admitted directly to the post natal ward in local neonatal units compared to special care units (p 0.03; CI 0.030.52) and neonatal intensive care units compared to local neonatal units (p 0.02; CI 0.028-0.211). The mean birth weight limit for direct PNW admission was significantly lower in neonatal intensive care units compared to special care units (p 0.011; CI 0.0280.211). There was no significant difference in mean birth weight for direct admission to PNW between SCUs and LNUs (p 0.23) or between LNUs and NICUs (p 0.07).

**Table 1:** Birth weight (BW) and gestational age (GA) limit for direct Postnatal Ward (PNW) admission

**Type of Neonatal Unit**	**Number responded**	**Mean (SD) and median (range) GA limit for directPNW admission**	**Mean (SD) and median (range) B W limit for direct PNW admission**
All Units	180	34.91 (0.71) wks	1.94 (0.2) kg
		35 (34–37) wks	2 (1.5-2.5) kg
SCU	49	35.19 (0.7) wks	1.99 (0.23) kg
		35 (34–37) wks	2 (1.7-2.5) kg
LNU	84	34.91 (0.67) wks	1.94 (0.20) kg
		35 (34–36) wks	2 (1.5-2.5) kg
NICU	47	34.61 (0.7) wks	1.87 (0.18) kg
		34 (34–36) wks	1.8 (1.5-2.5) kg

Comparing all units using a one way test of variance, the means for both birth weight and gestation remained significant with p values of 0.03 and 0.0005 respectively. 72/180 units (40%) had a paediatric nursery nurse on their post natal ward. When broken down by unit designation 35% (17/49) of SCUs, 38% (32/84) of LNUs and 48% (23/47) of NICUs had post natal ward nursery nurses. There was no statistically significant difference in relation to admission policy comparing mean birth weight and gestation, between units which had a nursery nurse on postnatal wards and those which did not. This persisted when data were analysed by unit designation (Tables 2 and 3).

**Table 2:** Gestational age (GA) limit for direct PNW admission in the presence or absence of a nursery nurse

**Unit designation**	**Mean (SD) GA cut off with Nursery Nurse present**	**Mean (SD) GA cut off without Nursery Nurse present**	**p value**
All units	34.83 (0.73) weeks	34.97 (0.71) weeks	0.21
SCU	35.24 (0.75) weeks	35.16 (0.69) weeks	0.73
LNU	34.81 (0.74) weeks	34.98 (0.62) weeks	0.27
NICU	34.57 (0.59) weeks	34.67 (0.86) weeks	0.64

**Table 3:** Birth weight (BW) limit for direct PNW admission in the presence or absence of a Nursery Nurse

**Unit designation**	**Mean (SD) BW cut off with Nursery Nurse present**	**Mean (SD) BW cut off without Nursery Nurse present**	**p value**
All units	1.91 (0.19) KG	1.96 (0.21) KG	0.14
SCU	1.99 (0.24) KG	1.99 (0.22) KG	0.99
LNU	1.93 (0.17) KG	1.95 (0.22) KG	0.75
NICU	1.82 (0.14) KG	1.94 (0.21) KG	0.058

## Discussion

Infants who are born late preterm represent the largest population among infants born <37 weeks gestation. At present, there is no routine data collection on the outcomes of late preterm infants in England. Although there are some international position statements with regards to care and monitoring of the late preterm infant [14], there is limited published data on what gestation and birth weight cut offs are used to decide whether these babies can be cared for in a mother-baby unit setting versus those requiring direct special care baby unit admission. This is the first survey which documents admission practices among all units in England and represents an important piece of data for ongoing surveillance of this group and for future service development and planning.

Until recently, the majority of research in relation to morbidity and outcome of preterm infants has focussed on infants born at extremes of prematurity [15]. This is not surprising given that this group is the most at risk among preterm babies. However, recent reviews have demonstrated that infants born late preterm are also at risk [16]. One of the issues facing clinicians who look after late preterm infants, is deciding which infants require admission to the neonatal or special care unit and which infants can be safely nursed on the post natal ward. There are clear advantages to keeping mothers and babies together. These include improved maternal and infant bonding and easier facilitation of breast feeding [17]. From the baby’s perspective, admission to the neonatal unit is frequently accompanied by intensive monitoring of vital signs, blood sugar and temperature. Late preterm infants are also more likely to undergo evaluations for suspected sepsis [18]. In some cases this level of care may delay discharge for certain babies.

Our study highlights that for the majority of units, care of some late preterm infants on the post natal ward is a consideration. In addition to the maternal and baby benefits, this practice also results in a significant cost saving as the daily cost of caring for infants admitted to neonatal intensive care and special care far exceeds that for infants and mothers nursed on the postnatal ward.

Based on our own local experience, any infant of gestation 35 weeks or more, whose birth weight is >1700 g and who is otherwise well, can be considered eligible for direct post natal ward admission. Regular departmental audits of this guideline have previously shown that approximately 76% of all late preterm infants who fulfil this criteria are admitted to the postnatal ward directly from the delivery suite with approximately 10% going on to require neonatal unit admission and a further 10% requiring readmission to hospital following discharge. We believe this strategy works well for our population of late preterm infants, though careful monitoring and follow-up after discharge is essential.

One of the limitations of this study is that other than asking about the presence or absence of paediatric nursery nurses on post natal wards, we did not establish why individual units adopt different direct post natal admission policies and how individual units came to establish their local guidelines. It is therefore difficult to explain why larger units appear to admit smaller babies born at earlier gestation to the post natal ward. The role of transitional care units on the postnatal ward requires further evaluation. We also acknowledge that there are many other providers of and factors influencing high quality infant care on the PNW that were not assessed in this study. These include midwifery staffing levels and training in addition to breast feeding advisors. Future studies may therefore concentrate on prospectively collected data on all late preterm infants who are directly admitted to the postnatal ward and the factors that influence their admission.

## Conclusion

This survey highlights different practices for direct postnatal ward admission of late preterm infants among neonatal intensive care and special care baby units in England. Further studies are needed to establish the factors influencing the difference in practice between units, and optimum immediate post natal care and long term follow-up for this growing population of preterm infants.

## Abbreviations

PNW: Post natal ward; SEND: Standardised electronic neontal database; SCU: Special care unit; LNU: Local neonatal unit; NICU: Neonatal intensive care unit; GA: Gestational age; BW: Birth weight.

## Competing interests

There are no competing interests, either financial or non-financial for any contributing author.

## Authors’ contributions

The contribution of each author is as follows: PF: Contributed to study design and data collection. Performed data analysis. Wrote the first draft of the manuscript and approved the manuscript as submitted. PA: Performed data collection. Participated in data analysis. RM: Contributed to study design and data collection. NA: Contributed to study design and overall supervision of the project. Participated in data analysis. All authors edited and approved the manuscript as submitted.

## Pre-publication history

The pre-publication history for this paper can be accessed here:

http://www.biomedcentral.com/1471-2431/14/150/prepub
